# Distinct ensembles in the noradrenergic locus coeruleus are associated with diverse cortical states

**DOI:** 10.1073/pnas.2116507119

**Published:** 2022-04-29

**Authors:** Shahryar Noei, Ioannis S. Zouridis, Nikos K. Logothetis, Stefano Panzeri, Nelson K. Totah

**Affiliations:** ^a^Neural Computation Laboratory, Istituto Italiano di Tecnologia, 38068 Rovereto, Italy;; ^b^Center for Mind/Brain Sciences, University of Trento, 38068 Rovereto, Italy;; ^c^Graduate Training Centre of Neuroscience, International Max Planck Research School (IMPRS), University of Tübingen, 72074 Tübingen, Germany;; ^d^Department of Physiology of Cognitive Processes, Max Planck Institute for Biological Cybernetics, 72076 Tübingen, Germany;; ^e^Division of Imaging Science and Biomedical Engineering, University of Manchester, M13 9PL Manchester, United Kingdom;; ^f^ International Center for Primate Brain Research (ICPBR), 201602 Shanghai, China;; ^g^Department of Neural Information Processing, University Medical Center Hamburg-Eppendorf, 20251 Hamburg, Germany;; ^h^Helsinki Institute of Life Science, University of Helsinki, 00014 Helsinki, Finland;; ^i^Faculty of Pharmacy, University of Helsinki, 00014 Helsinki, Finland

**Keywords:** ensemble, cortical state, neuromodulation, locus coeruleus, cortex

## Abstract

The noradrenergic locus coeruleus (LC) is a controller of brain and behavioral states. Activating LC neurons en masse by electrical or optogenetic stimulation promotes a stereotypical “activated” cortical state of high-frequency oscillations. However, it has been recently reported that spontaneous activity of LC cell pairs has sparse yet structured time-averaged cross-correlations, which is unlike the highly synchronous neuronal activity evoked by stimulation. Therefore, LC population activity could consist of distinct multicell ensembles each with unique temporal evolution of activity. We used nonnegative matrix factorization (NMF) to analyze large populations of simultaneously recorded LC single units in the rat LC. NMF identified ensembles of spontaneously coactive LC neurons and their activation time courses. Since LC neurons selectively project to specific forebrain regions, we hypothesized that distinct ensembles activate during different cortical states. To test this hypothesis, we calculated band-limited power and spectrograms of local field potentials in cortical area 24a aligned to spontaneous activations of distinct LC ensembles. A diversity of state modulations occurred around activation of different LC ensembles, including a typical activated state with increased high-frequency power as well as other states including decreased high-frequency power. Thus—in contrast to the stereotypical activated brain state evoked by en masse LC stimulation—spontaneous activation of distinct LC ensembles is associated with a multitude of cortical states.

## Significance

Brainstem locus coeruleus (LC) noradrenergic neurons produce an arousal-related state characterized by a broadband increase in high-frequency oscillations. This perspective was built upon electrical or optogenetic stimulation that artificially activates LC neurons synchronously. This has led to the conceptual model that LC activation is associated with a single cortical state. Here, we show that natural, spontaneously occurring LC single-unit activity consists of ensembles with largely nonoverlapping activation dynamics. Spontaneous activations of different LC ensembles are associated with different cortical states. Our results suggest that the role of the LC in controlling a single type of cortical state associated with arousal is an oversimplification. Instead, ensembles of LC neurons may control a diverse multitude of cortical states.

Flexible behavior is associated with transitions across diverse cortical states. For example, various states of wakefulness, perceptual ability, and behavioral activity are associated with different cortical local field potential (LFP) and electroencephalogram (EEG) states, each with its own clear spectrotemporal pattern of neural oscillations (1–3). Behavioral state transitions, such as waking from sleep or entering a state of heightened stress and reacting more quickly to stimuli, are associated with cortical state transition. These changes are not necessarily driven by external stimuli. Instead, cortical state can be controlled by factors internal to the organism (e.g., sleep need and perceived stress) and therefore arise from self-organized neuronal interactions. It remains unclear exactly which interactions among neurons control cortical states.

Cortical states are mediated, at least in part, by the brainstem nucleus the locus coeruleus (LC). The LC releases norepinephrine to modulate neuronal excitability (4–7). Noradrenergic neuromodulation of cortical state has been studied using electrical or optogenetic stimulation. Such stimulation evokes highly synchronous activation of many LC neurons because this brainstem nucleus (in rats) contains ∼1,600 neurons tightly packed into a small volume of ∼200 × 500 × 1,000 μm (8, 9). Even a low stimulation current (0.03 to 0.05 mA) pulse evokes spiking up to 400 μm from the stimulation site in the rat LC (10) and thus synchronously activates many LC neurons. Such en masse LC population activation evokes an activated cortical state characterized by an increase in high-frequency power and a decrease in low-frequency power, regardless of whether the subject is anesthetized or not (10–13). Critically, it is still unknown how spontaneous population activity in the LC, as opposed to LC activity evoked by stimulation, relates to cortical states.

Although noradrenergic neuromodulation of cortical state has largely been studied using external stimulation of LC, cortical state emerges from spontaneously occurring internal neuronal interactions. Spontaneous LC neuronal population activity has been traditionally thought to be highly synchronous (14–19), akin to the en masse population activity evoked by LC stimulation. However, this standard view might not describe spontaneous LC activity accurately. Graph theoretic analysis of time-averaged cross-correlations among pairs of spontaneously active LC neurons in anesthetized rats showed sparse yet structured pairwise correlations that are not characteristic of highly synchronous population activity (20). This suggests that LC population activity potentially consists of multicell ensembles that become active at different times. Importantly, however, pairwise graph theoretic analyses were based upon time-averaged measures (20) and could not detect multicell ensembles or resolve ensemble activity over time.

Here, we used nonnegative matrix factorization (NMF) (21) to analyze large populations of simultaneously recorded single units in the rat LC. NMF decomposes the time course of population activity into often-recurring population firing patterns, from which ensembles of neurons that often fire together can be identified. Critically, NMF also quantifies how strongly a population firing pattern or an ensemble was active at any given time. We applied NMF to simulated synthetic spike trains and found that NMF detects the precise neuronal composition and activation time courses of each ensemble. In comparison, graph theoretic analysis of time-averaged correlations could not detect ground truth ensemble activity in these synthetic spike trains. Importantly, unlike graph theoretic analyses operating on time-averaged pairwise correlations of neuronal activity, NMF resolved the activity of discrete LC ensembles over time. This allowed us to demonstrate that LC population activity consists of discrete LC ensembles each with its own evolution of activity over time.

Given that LC neurons selectively project to specific forebrain regions, we investigated how individual LC ensembles are related to different cortical states. One possibility is that different ensembles may simply evoke the stereotypical activated state (as observed after LC stimulation) but with different ensembles being associated with variations in cortical activated state duration or power magnitude. A second and more intriguing possibility is that distinct LC ensembles activate during a wide range of cortical states that have different spectral signatures. This would support a new perspective that LC ensembles may contribute to the diverse set of cortical states that characterize flexible behavior.

Since our methods allowed tracking of the spontaneous temporal dynamics of individual LC ensembles, we could relate ongoing LC ensemble dynamics to cortical state dynamics. We calculated the cortical area 24a LFP band-limited power (BLP) and power spectra in a window aligned to the spontaneously occurring activation times of each LC ensemble. In contrast to the standard view that LC population activity evokes a stereotypical activated cortical state, we observed heterogenous cortical states with different spectral and temporal properties that depended on which LC ensemble was active.

## Results

We recorded many LC single units simultaneously (range: 5 to 34 units; average: 19 units; *n* = 15 male rats) under urethane anesthesia using a 32-electrode silicon probe confined to the core of the LC nucleus. Probe location was verified histologically in coronal tissue sections. Neuronal identity was confirmed at the end of the experiments using intraperitoneal injection of the α-2 agonist clonidine, which inhibited spiking on all electrodes. Spikes recorded from outside the LC core would not have been inhibited due to the lack of α-2-adrenergic receptors in nearby brain structures (22). We simultaneously recorded cortical LFP (8-kHz lowpass–filtered) from cortical area 24a (anterior cingulate cortex) (23) using a tungsten electrode in 9 of the 15 rats.

### NMF Detects the Neuronal Composition and Activation Times of Ensembles.

It is currently unknown whether the spontaneous LC population activity that we recorded is composed of ensembles (subsets of simultaneously active neurons) and how ensemble activity changes over time. A graph theoretic community detection analysis of time-averaged pairwise correlations in the LC demonstrated groups of units linked by pairwise time-averaged correlations (20). This result is compatible with the possibility that spontaneous LC activity consists of ensembles. However, this analysis may fail to detect ensembles if their interaction is based on higher-order interactions between larger groups of neurons that cannot be linearly decomposed into pairwise correlations (24). Moreover, such time-averaged analysis cannot identify the times at which individual ensembles are active, which is needed for studying the relationship between spontaneous LC ensemble activity and ongoing cortical state. Thus, we need a methodology to detect ensembles and measure their activation times. Among available alternatives (25), we chose to use NMF because it offers several advantages given the nature of spike trains (26, 27). First, NMF linearly decomposes spike trains based only on nonnegative constraints on the detected firing patterns and their strength of activation over time. This nonnegativity constraint is a minimal and biologically grounded assumption for spike trains. Second, unlike decompositions that are based on orthogonality constraints (28), NMF has been designed and proven to work well even when different ensembles are nonorthogonal because they activate partly overlapping in time or because some neurons participate in multiple ensembles. We first use synthetic spike trains with ground truth ensemble activity patterns to demonstrate how NMF goes deeper than the graph theory analysis previously applied to LC data (20) to identify recurring firing patterns, extract ensembles from these firing patterns, and determine the ensemble activation dynamics over time.

**Fig. 1.**
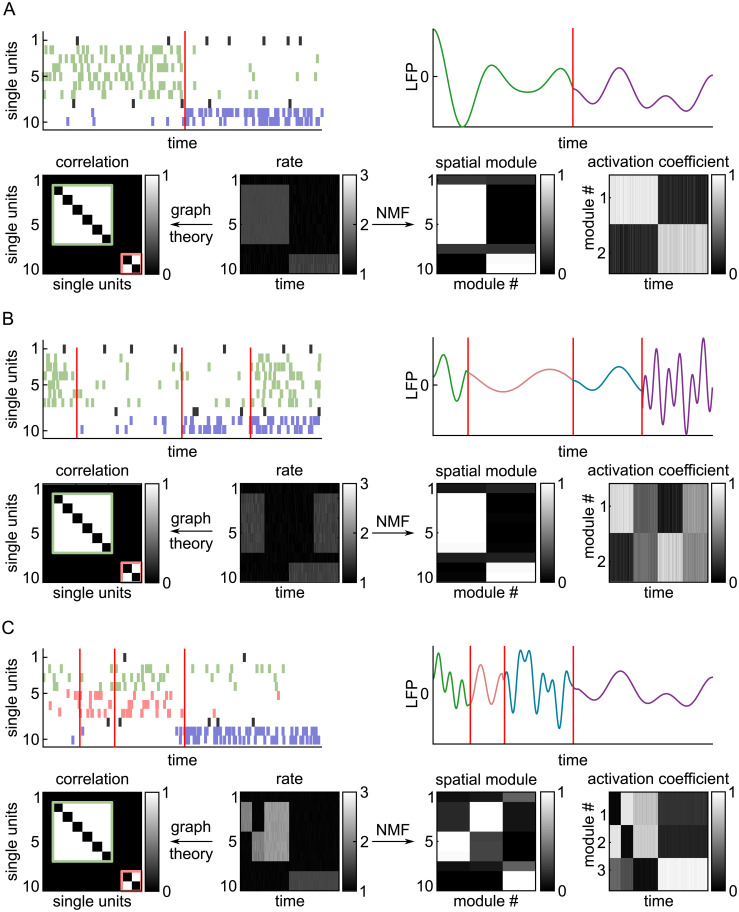
Comparison of NMF versus graph theoretic analysis for detecting ground truth ensemble dynamics from synthetic spike trains. Three different simulated spike rasters were generated to compare the NMF method with the graph theoretic method. (*A*) Scenario 1 (two ensembles active at distinct times). (*B*) Scenario 2 (two ensembles active at distinct and overlapping times). (*C*) Scenario 3 (three ensembles active at distinct and overlapping times). *Top Left*, Simulated spike rasters of 10 single units. The spikes belonging to different ground truth ensembles are in different colors. The red lines indicate transitions in the population activity. *Top Right*, Simulated ongoing cortical LFP with different cortical states plotted in different colors. *Bottom Middle*, Binned spike rates of each unit. *Bottom Left*, Neuronal groups detected by graph theoretic analysis of time-averaged pairwise correlations. White indicates a significant time-averaged correlation for the pair defined by each axis. Groups of coactive neurons are indicated by the green and red boxes ( *SI Appendix*, *Materials and Methods* ). *Bottom* *Right*, NMF decomposition into the spatial modules and their activation time courses.

Fig. 1 illustrates how NMF works using three different hypothetical scenarios of simulated spike trains. Each scenario consisted of simulated spike trains from 10 single neurons. The ground truth ensemble dynamics were different in each scenario. In the first scenario (Fig. 1*A*), two ensembles (ensemble 1: units 2 to 7; ensemble 2: units 9 to 10) were strongly activated at distinct times. In the second scenario (Fig. 1*B*), the same two ensembles were strongly activated at distinct times in the first and third part of the simulation, were inactive in the second part, and then were simultaneously coactivated in the last part of the simulation. Finally, in the third scenario (Fig. 1*C*), we simulated three distinct ensembles (ensemble 1: units 2 to 4; ensemble 2: units 5 to 7; ensemble 3: units 9 and 10). Ensembles 1 and 2 had different temporal activation patterns with often only one of the two ensembles being active, but they also had a period in which they were both strongly coactive.

NMF decomposes the rate matrix containing the population vectors at all time points into a sum of *K* nonnegative spatial modules, each of which is multiplied by a nonnegative activation coefficient. Formally, a spatial module is a vector (one entry per neuron) specifying the relative strength of firing of each neuron (26, 27). A spatial module may be thought of as an often-recurring population firing pattern. Thresholding these spatial module values defines the ensemble of specific single units that were significantly active within each module. For example, in Fig. 1 *A* and *B*, NMF identifies spatial module 1 as a population firing pattern consisting of high activity of units 2 to 7 and low activity for the other units. The high (thresholding-crossing) module values identify units 2 to 7 as the ensemble 1 that was activated in module 1. For each spatial module, its activation coefficient at any given time describes how strongly the spatial module (and thus the ensemble of coactive units within it) is recruited. By thresholding the activation coefficients, we detected the times of occurrence of each spatial module (population firing pattern) and of the associated ensemble. These will be referred to as “ensemble activation times.”

The number of spatial modules (*K*) was determined, both for these synthetic data and for actual LC population recordings, based on two criteria. First, the chosen *K* explained a high amount of variance in the data with the fewest possible spatial modules. *K* was in the “elbow” region of the reconstruction error, such that a higher *K* would have given diminishing returns in data reconstruction accuracy when plotted as a function of the possible number of spatial modules ( *SI Appendix*, Fig. S1). Second, the selected *K* yielded stable spatial modules regardless of the random initialization of the procedure ( *SI Appendix*, *Materials and Methods* ).

We previously used graph theoretic analysis of time-averaged cross-correlations to detect correlated activity among multiple pairs of LC neurons (20). To understand how it compares with NMF, we performed this previous analysis on the synthetic spike trains. Despite the difference in ensemble dynamics, ensemble composition, and number of ensembles across scenarios, graph theory detected the same two groups of strongly correlated units in each scenario (Fig. 1). The temporal activation patterns of these neuronal groups cannot be obtained because graph theoretic analysis uses time-averaged activity. Therefore, while graph theoretic analysis could not identify the precise neuronal composition of ensembles or their activation dynamics, NMF identified these ensemble properties correctly in all three simulated scenarios.

### Spontaneous LC Population Activity Consists of Distinct Ensembles with Largely Nonoverlapping Activation Dynamics.

We assessed whether LC population activity consists of ensembles using NMF on the population matrix containing the time-dependent binned spike count vectors of simultaneously recorded single units independently for each rat. We binned activity in 100-ms sliding windows, which is the time scale capturing most of the synchrony among LC single-unit pairs (20).

**Fig. 2.**
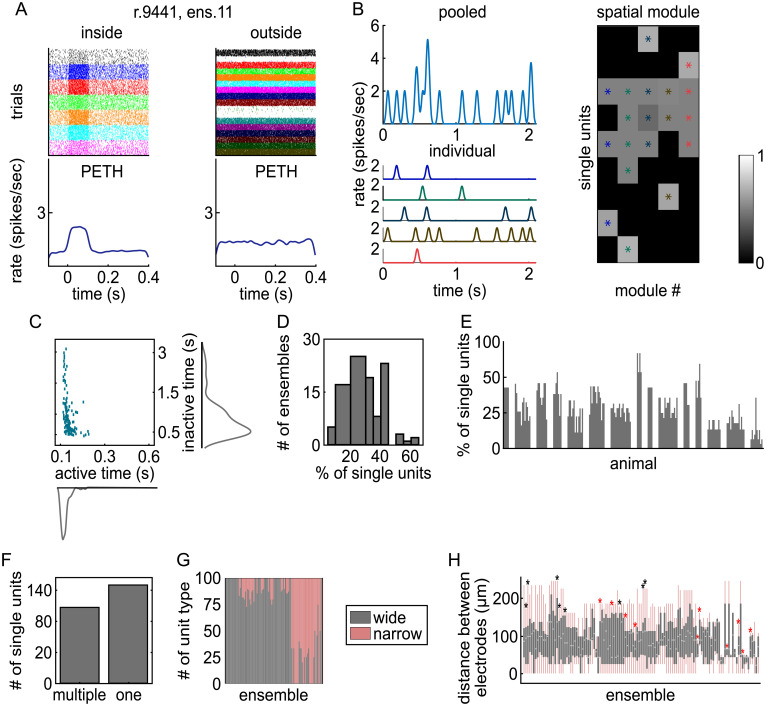
Characteristics of LC ensembles. (*A*) The spike rasters and PETHs are shown for one exemplar ensemble. *Left*, Spike rasters of the single units inside the ensemble aligned to the ensemble activation times (*t* = 0 s). *Right*, The same for other simultaneously recorded single units that were not assigned to this ensemble. Each ensemble activation event is a “trial” in the spike raster. The PETHs show the spike rate averaged over units in the ensemble and then averaged over all detected ensemble activation events. (*B*) An example LC recording in which NMF found five ensembles among nine single units. *Upper Left*, Population activity pooled by summing spike rate across all simultaneously recorded single units. *Lower Left*, Activation coefficients of five individual ensembles in different colors. *Right*, Spatial module values for each single unit. A threshold was applied to these values to identify which single units were significantly active in each spatial module firing pattern. Units that crossed the threshold are marked with an asterisk and form an ensemble (each color is an ensemble). (*C*) The scatter plot shows the average ensemble active times versus inactive times along with the corresponding histograms. (*D*) The distribution of ensemble sizes is plotted. The values are the percentage of simultaneously recorded single units that were assigned to an ensemble. (*E*) Each bar reports the percentage of single units assigned to that ensemble. Bars are grouped by rat. Note that a single unit can be part of more than one ensemble. (*F*) Number of single units participating in one ensemble or multiple ensembles. (*G*) The percent of each unit type (wide or narrow) making up each ensemble is plotted across ensembles. Rats in which only one single unit type was recorded are not included in this plot. (*H*) Box plots show the distribution of the distances between each pair of single units within each ensemble. Ensembles with only two single units were excluded. The light gray line shows the median, the gray box shows the interquartile range, and the pink line shows the full range of the data. Asterisks indicate the ensembles with pairwise distances between units within the ensemble having a more diffuse (black asterisks) or more constrained (red asterisks) spatial organization than expected from a random spatial distribution.

NMF found 146 ensembles of coactive LC single units from 15 rats. Fig. 2*A* shows an example ensemble from a recording of 24 single units. The rasters show 7 single units that spontaneously coactivated as an ensemble for a transient period of ∼100 ms as well as the unchanging baseline-level activity of the remaining 17 units not assigned to that ensemble. The corresponding perievent time histograms (PETHs) describe the time course of average spike rate for single units within the ensemble and the single units not assigned to that ensemble. Around the time of ensemble activation (at *t* = 0 s), units assigned to this example ensemble coactivated, whereas the activity of units outside the ensemble did not change. We verified over the entire dataset that single units not assigned to an ensemble did not increase their spike rate when the ensemble was active, whereas units assigned to the ensemble did ( *SI Appendix*, Fig. S2).

Fig. 2*B* shows an example in which LC population activity was decomposed into five distinct ensembles. The ensembles were active in most cases at different times, but occasionally more than one ensemble was simultaneously active (e.g., brown and red lines at *t* = 0.5 s). Reconstructing the total population firing rate as a function of time through NMF decomposition (i.e., essentially summing up the activation time courses across the five ensembles) returned a close approximation of the pooled population spike rate. This example suggests that LC neuronal population activity is composed of distinct ensembles that activate at largely nonoverlapping times.

Most ensembles were only transiently active for 100 ms (Fig. 2*C*). The median ± SE of the median activation duration over all ensembles was 114 ± 8 ms (*n* = 146 ensembles). The average PETH across all ensembles showed that activation decayed sharply around 100 ms after activation onset ( *SI Appendix*, Fig. S2). However, distinct ensembles were quiet for a wide variety of durations between these brief activations (Fig. 2*C*). The duration of the inactive periods was highly variable across ensembles (median ± SE of the median = 611 ± 295 ms). These findings are compatible with the view that distinct ensembles have different spontaneous dynamics.

LC neurons spontaneously fire bursts of action potentials, typically with an interspike interval of <100 ms (29–33); therefore, we also assessed whether LC ensemble activation times could occur in a burst pattern. We calculated auto-correlograms of the time course of the ensemble activation coefficients ( *SI Appendix*, Fig. S3). We found almost no interactivation intervals in the 100 ms after ensemble activation. The most prominent characteristic of ensemble activity dynamics was a tendency for rhythmic ensemble activation at 500 to 700 ms. Thus, burst patterns did not occur in LC ensemble activation dynamics.

Ensembles were comprised of relatively small subsets of single units. On average, 27% of single units were active in ensembles relative to the total number of simultaneously recorded single units in each rat (Fig. 2*D*). Ensemble size ranged from 6 to 62% of the simultaneously recorded single units (Fig. 2*E*). We assessed whether ensembles consisted of disjoint sets of single units or overlapping single units. Out of 285 single units, 115 participated in multiple ensembles (40.4%), 149 participated in only one ensemble (52.3%), and 21 did not participate in any ensemble (Fig. 2*F*).

Ensembles preferentially consist of one type of LC single unit (narrow or wide [20]), defined by their extracellular waveform shape (Fig. 2*G*). We assessed if the proportion of each unit type participating in each ensemble was statistically different from what would be expected if ensembles were formed by units taken by random resampling regardless of type. For all rats, the hypothesis that ensembles are formed by combining units regardless of their type was rejected (*P* < 0.05).

We examined the spatial distribution of units within the ensembles. We assessed (by randomly shuffling the recorded location of units) whether the median distance between unit pairs within the ensemble differed from a random spatial organization. Location was defined as the electrode on the array that recorded the maximal spike amplitude (averaged across all spikes). Only a small proportion of ensembles (18 out of 121 ensembles with more than two units) had a median distance between unit pairs that differed from the one expected by a random spatial organization (at *P* < 0.05 with the null hypothesis distribution computed with 100 shuffles; Fig. 2*H*). Finally, for each ensemble, there was no difference in the distribution of distances between unit pairs belonging or not belonging to the same ensemble (Wilcoxon’s rank sum test with false discovery rate correction for 121 ensembles; lowest *P* value was *P* = 0.1434). These results are consistent with a nontopographical diffuse arrangement of LC neurons spontaneously firing as an ensemble.

### LC Ensembles Are Temporally Distinct and Are Sparsely Active.

**Fig. 3.**
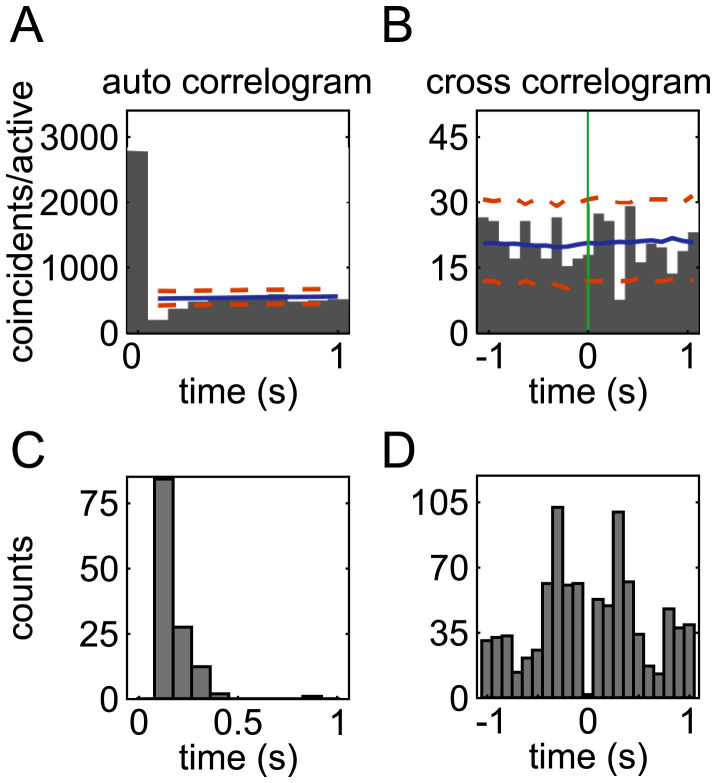
Ensemble auto-correlogram and ensemble pair cross-correlogram troughs. (*A*) The histogram plots the auto-correlogram (time binning of 100 ms) of the activation time course of an example ensemble (ensemble 5 from rat 2591). A trough in the auto-correlogram denotes postactivation inhibition. Significant troughs were defined as those that had auto-correlogram values below the first percentile (lower dashed orange line) of the surrogate distributions of correlogram values computed by randomly jittering ensemble activation times. The solid blue line shows the average of the surrogate correlograms. (*B*) The histogram plots the cross-correlogram of the activation time course of an example ensemble pair (ensembles 9 and 11 from rat 2632). The activation time of the reference ensemble is at *t* = 0 s (green line). A frequent silence of one ensemble 400 ms after the other spontaneously activated is shown by the significant trough. Plotting conventions are as in *A*. Significant troughs of cross-correlograms were computed with the same method used for the auto-correlograms. (*C*) Histogram of the number of significant auto-correlogram troughs as a function of the time lag. (*D*) Histogram of the number of significant cross-correlogram troughs across time lags.

Before considering how the activation of LC ensembles relates to cortical states, it is important to further characterize the relative dynamics across ensembles in order to form hypotheses about whether different ensembles have sufficiently distinct dynamics to potentially produce ensemble-specific cortical states. We first characterized when the activation of an ensemble makes it less likely that the same or another ensemble is active at some other time. We computed auto-correlogram and cross-correlograms of the time course of the ensemble activation coefficients and detected significant troughs in the correlograms (Fig. 3 *A* and *B*). A trough in the auto-correlogram occurred in 62% of the ensembles (90 out of 146). For these ensembles, the decreased spiking was most frequent at a 100-ms delay after ensemble activation but could occur as late as 300 ms after ensemble activation (Fig. 3*C*). We found that 44% of ensemble pairs (348 out of 790) had a significant cross-correlogram trough. Cross-correlogram troughs were most frequent after a delay of ±300 ms but covered a wide range of times lasting up to 1 s (Fig. 3*D*). The times at which troughs were observed match well with the wide range of pauses apparent in ensemble activity shown in Fig. 2*C*. These pauses in the activity of single ensembles and in the relative timing between ensemble pairs produce sparse activations of distinct LC ensembles and provide evidence that different ensembles have distinct activation dynamics.

### LC Ensembles Can Be Synchronously Active.

Although our results demonstrate that LC ensembles are distinct from one another and activate with largely nonoverlapping time courses that contain numerous highly variable firing pauses, it does not preclude the possibility that LC ensembles coactivate. Examples of this in LC data can be seen in Fig. 2*B* (e.g., brown and red lines at *t* = 0.5 s). The occurrence of ensemble coactivation is important to quantify because such ensemble population activations would be more like the en masse firing evoked in LC stimulation studies that have been used to define the role of the LC in modulating cortical state (10–13).

**Fig. 4.**
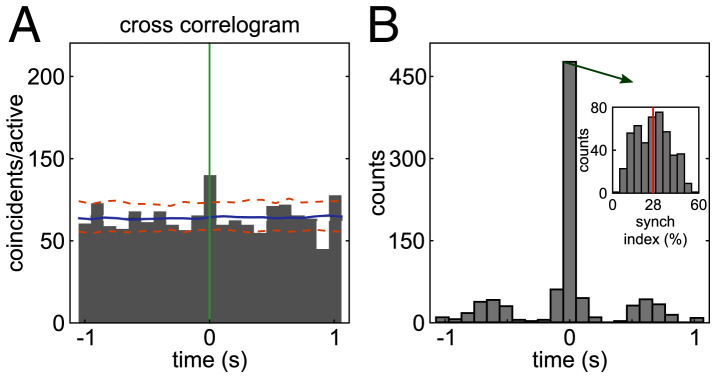
Peaks of cross-correlograms between the activation times of ensemble pairs. (*A*) Histogram of the cross-correlogram (time bin 100 ms) of the activation time course of an example ensemble pair (ensembles 1 and 2 from rat 1052) with a significant zero lag cross-correlation peak. The activation time of the reference ensemble is at *t* = 0 s (green line). Significant peaks were defined as those that had cross-correlogram values above the 99th percentile (upper dashed orange line) of the surrogate distributions of correlogram values computed by randomly jittering ensemble activation times. The solid blue line shows the average of the surrogate correlograms. (*B*) The histogram shows the number of ensemble pairs with a significant cross-correlogram peak as a function of time lag. Most peaks occur at zero lag. *Inset*, histogram of synchrony index values for the 420 ensemble pairs that had significant zero lag cross-correlogram peaks.

We computed the cross-correlograms between the activation time courses of each ensemble pair and quantified both the number of ensemble pairs with significant (positive) peaks of cross-correlation and the lag of the cross-correlogram highest peak for those ensembles. We found that 506 of 790 ensemble pairs (64%) had significant cross-correlogram peaks. An example significant peak at zero lag is shown in Fig. 4*A*. We found that most of the significant peaks occurred at zero lag (Fig. 4*B*). Out of 506 ensemble pairs with significant cross-correlation peaks, 420 (83%) had a zero lag peak. In the overall population of 790 ensemble pairs, synchronous coactivation was observed among 53% of ensemble pairs. This result suggests that although ensembles are distinct, a large proportion of ensemble pairs can coactivate.

To measure how frequently ensemble pairs synchronously coactivate, we calculated a zero lag synchronization index for each of the 420 ensemble pairs with a significant zero lag cross-correlogram peak. This index quantifies the percentage of activation instances of an ensemble that were zero lag coactivations with another ensemble. The average synchronization index was 28% with a range of 3 to 59% (Fig. 4*B*, *Inset*, *n* = 420 ensemble pairs). Importantly, the remaining 47% of ensemble pairs (370 out of 790) never coactivated (no zero lag peak in the cross-correlogram). Overall, and contrary to the standard view that the LC neuronal population fires en masse with a high level of synchrony, these analyses show that LC ensembles have ensemble synchronous coactivations, but this happens for only approximately half of the ensembles that synchronously coactivate on average approximately one-quarter of the time. Overall, for the most part, ensemble dynamics are nonoverlapping.

### Spontaneous Activation of Distinct LC Ensembles Is Associated with Different Cortical States.

The largely nonoverlapping dynamics of individual LC ensembles may enable distinct ensembles to evoke different cortical states. Alternatively, all ensembles may promote the stereotypical activated state often imputed to LC activation (1–3, 10–13). We disambiguated between these two possibilities by characterizing cortical state around the time of spontaneous LC ensemble activations.

We aligned the cortical area 24a LFP to the times of spontaneous activation of individual LC ensembles. For this analysis, NMF identified 89 ensembles in nine rats from which cortical LFPs and LC population activity were simultaneously recorded. We calculated both the LFP spectrogram and the BLP in five canonical frequency bands ( θ, 4−8 Hz;α, 8−12 Hz;β,12−30 Hz;γ, 30−70 Hz; high γ, 70−150 Hz ), analyzing data from a 900-ms window aligned to spontaneous activation of each LC ensemble. This window began 400 ms before the beginning of ensemble activation, ran through a fixed 100 ms meant to approximate the ∼100-ms average duration of ensemble activation, and continued for 400 ms after the end of ensemble activation. This window was chosen for two reasons. First, it provided a good tradeoff between temporal and spectral resolution. Second, our previous analyses of cross-correlations and durations of activation and activity pauses show that it is unlikely that multiple ensembles were coactive during this window (Figs. 2*C* and 4*B*). Therefore, this window ensured that changes in the cortical LFP spectrum were predominantly related to activation of an individual ensemble in the LC. We averaged the BLP and the spectrogram for each ensemble over all instances of its activation. To compare spectrograms and BLP across ensembles, we normalized them using a spectral modulation index, which quantifies the relative changes of power around the time-averaged power in each frequency bin ( *SI Appendix*, *Materials and Methods* ).

**Fig. 5.**
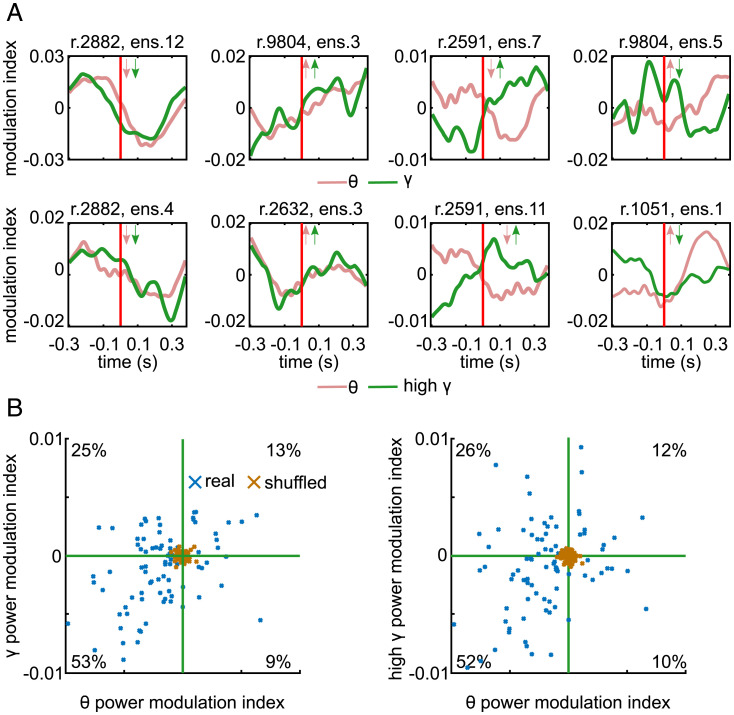
Modulations of cortical LFP BLP aligned to spontaneous activation of LC ensembles. (*A*) Examples of BLP (calculated using the spectrogram modulation index) for different ensembles. The *Upper* row shows θ band (red) and γ band (green) cortical BLP in a window around ensemble activation onset (marked by a vertical red line) for four example ensembles. *Inset* arrows indicate whether time-averaged power in each band increased or decreased after the ensemble was active relative to the window prior to ensemble activation. The *Lower* row shows the θ  band (red) and high γ band (green). Plotting conventions are identical for both rows. (*B*) Scatter plots show the time-averaged BLP 400 ms after spontaneous ensemble activation. Each blue data point is an ensemble (*n* = 89). *Left*, θ band versus γ band. *Right*, θ band versus high γ band. The percentages indicate the percent of ensembles (blue crosses) in each quadrant. The green line indicates 0 on each axis. Brown crosses represent surrogate (shuffled) BLP modulations obtained by recomputing BLPs after randomly shuffling the times of ensemble activations.

Visual inspection of individual examples of BLP aligned to LC ensemble activation revealed diverse patterns of modulations in cortical BLP around the ensemble activation time depending on which ensemble was active (Fig. 5*A*). Importantly, BLP modulations averaged over the 400 ms after spontaneous activation of each ensemble were larger than those obtained when recomputing surrogate BLP modulations after randomly shuffling the ensemble activation times (Fig. 5*B*). The scatter plots showing the changes in BLP revealed a structure within the large diversity of power changes associated with individual ensembles (Fig. 5*B*). After spontaneous activation of approximately half of the ensembles, there was a relative decrease of cortical power both in the low ( θ ) and in the high ( γ, high γ ) frequencies with respect to preactivation power. However, for approximately one-quarter of the ensembles, there was a relative decrease of low-frequency power and an increase of high-frequency power similar to the cortical activated state observed after external stimulation of the LC (10–13). Relative increases of both low-frequency and high-frequency power as well as relative increases of low-frequency power but decreased high-frequency power were also observed after activation of smaller proportions of ensembles.

**Fig. 6.**
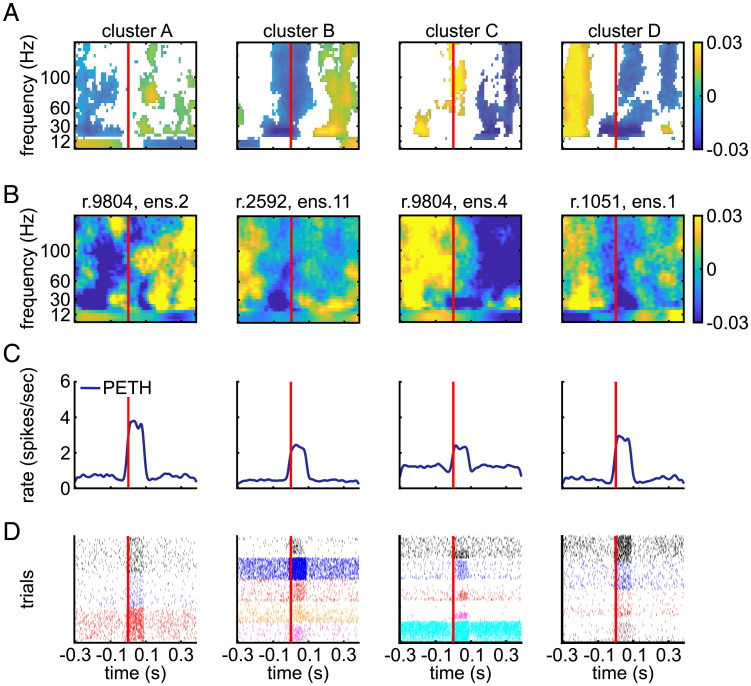
Modulations of cortical LFP spectrograms aligned to spontaneous activation of LC ensembles. (*A*) The average across all spectrograms within each cluster aligned to ensemble activation time (red line). Significant modulations are in color, and nonsignificant values are white. The significance of spectrogram modulations of cluster centers was computed as described in *Materials and Methods*. (*B*) The ensemble activation–aligned spectrogram for an example ensemble from each cluster. Plotting conventions are the same as in *A*. (*C* and *D*) For each example ensemble in *B*, the PETH of its population spike rate (*C*) and the spike rasters of single units in the ensemble (*D*) are plotted around spontaneous activation of the ensemble (red lines). For all graphs, we plotted data in a periensemble activation window from 300 ms before to 400 ms after ensemble activation onset because the spectrograms were estimated in sliding windows whose centers all fell within this window.

Similar trends were found when considering individual examples of spectrograms computed around ensemble event activations (Fig. 6 and *SI Appendix*, Fig. S4). Considering spectrograms is useful because it gives more detailed spectral information than BLP. To capture typical trends in the cortical spectrograms (from 4 Hz to 150 Hz) across LC ensembles (*n* = 89), we clustered the spectrograms calculated in the same periensemble activation window considered above for BLP. This clustering analysis found four predominant spectrogram types with different trends in spectrotemporal modulations, which we term clusters A, B, C, and D. We chose four clusters by first varying the putative number of clusters from 1 to 22 and quantifying the diminishing returns of adding each additional cluster ( *SI Appendix*, Fig. S5). Critically, only one of these spectrogram types (cluster A, Fig. 6*A*, associated with activation of 28% of the ensembles) had a spectral change characterized by a relative increase in high-frequency power and a relative decrease of low-frequency power from before to after LC ensemble activation, which resembled the stereotypical activated cortical state observed after electrical or optogenetic stimulation of the LC (10–13).

Another spectrogram type (cluster B, associated with activation of 23% of the ensembles) was characterized by an overall relative increase in oscillatory power across all frequencies after ensemble activation as well as a broadband decrease just prior to and during ensemble activation. Two others (clusters C and D, associated with activation of 24% and 25% of the ensembles, respectively) showed a relative decrease of power across all frequencies above the θ band. Cluster C was also accompanied by a brief increase in high-frequency power during ensemble activation.

The patterns seen with the clustering of the spectrograms in Fig. 6*A* recapitulate reasonably well the major different behaviors found across all individual ensembles as shown by the distribution of relative modulations of BLP in Fig. 5. For instance, half of ensembles show a relative decrease of BLP power across all frequencies after ensemble activation (lower left quadrants in Fig. 5*B*), matching well the finding of two out of four spectrogram clusters with a generalized relative power decrease after ensemble activation (Fig. 6*A*). It is important to note that the four spectrogram clusters do not indicate that those were the only kind of state transitions happening, rather they represent the centroids of the most common trends recapitulating predominant behaviors.

**Fig. 7.**
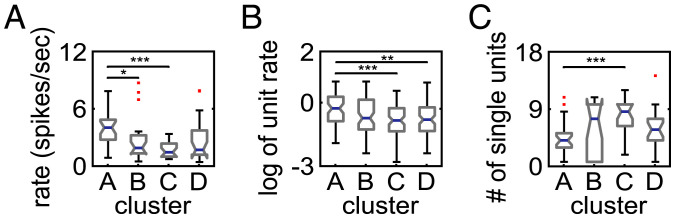
The firing properties of LC ensembles associated with each cortical spectrogram cluster. (*A*) Box plots of ensemble activation strength in each spectrogram cluster calculated using the peak of the ensemble population firing rate PETH. (*B*) Box plots of ensemble activation strength in each spectrogram cluster calculated using the single-unit averaged firing rate PETH. Due to the skewness of the single-unit firing rate distributions, we plot log-transformed data. (*C*) Box plots of the number of single units within the ensembles for the different spectrogram clusters. Significance is indicated by post hoc tests: *P < 0.05; **P < 0.01; ***P < 0.001.

We next assessed whether firing properties differed between distinct groups of ensembles associated with the four cortical spectrogram clusters (Fig. 7). We first characterized the strength of ensemble activation and found that it differed across the ensembles associated with the four spectrogram clusters. The population spike rate was averaged across all ensemble activation events and all single units in the ensemble. The peak of the resulting population spike rate PETH was used to characterize the activation strength of the ensemble. We found that the median activation strength across ensembles associated with each cortical spectral cluster differed (Kruskal–Wallis test, *P* = 0.0003, $\omega^{2}$ = 0.9634, $\chi^{2}$ = 18.8899, *n* = 89 ensembles). Post hoc tests showed that cluster A differed from clusters B and C (Fig. 7*A*). We also examined the ensemble activation strength by first averaging across ensemble activation times to obtain a PETH for each single unit separately and then averaging across single units. Activation strength of the ensemble was the peak of the single unit averaged PETH. The median activation strength across ensembles in each cortical spectral cluster type again differed (Kruskal–Wallis test, *P* = 0.0008, $\omega^{2}$ = 0.9869, $\chi^{2}$ = 16.8530, *n* = 89 ensembles). Activation strength differed between clusters A and C and clusters A and D (Fig. 7*B*). Regardless of how ensemble activation strength was assessed (Fig. 7 *A* and *B*), ensembles associated with spectral cluster A were more strongly active than ensembles associated with other clusters. Finally, we examined whether the size of the ensemble differed across cortical spectral clusters. The median number of units across ensembles differed across clusters (Kruskal–Wallis test, *P* = 0.0012, $\omega^{2}$ = 0.9618, $\chi^{2}$ = 15.8187). Post hoc tests showed that cluster A had fewer units than cluster C (Fig. 7*C*). In summary, ensembles associated with cluster A spectra (“activated state”) had smaller size and stronger spontaneous activations than other ensembles.

### Synchronous Spontaneous Activation of a Larger Pool of LC Ensembles Results in a More Homogeneous Cortical State.

**Fig. 8.**
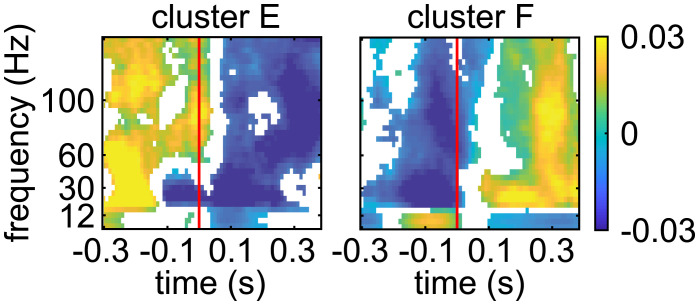
Modulations of cortical LFP spectrograms aligned to spontaneous coactivation of LC ensemble pairs. The spectrograms are aligned to the coactivation times of ensemble pairs that had a significant zero lag cross-correlation peak. The onset of ensemble pair coactivation is indicated by the red line. The resulting spectra clustered into two types. Each plot shows the average spectrogram across all ensemble pairs associated with each cluster. Only significant changes in the power spectrum are plotted in color, and nonsignificant modulations are white. Colors correspond to spectral modulation index values, as in Fig. 6 *A* and *B*.

These data demonstrate a relationship between distinct LC ensembles and cortical states. This finding stands in marked contrast to the stereotypical activated state evoked by electrical or optogenetic stimulation that synchronously activates LC neurons. Therefore, we predicted that when several LC ensembles are coactive (a situation that may resemble more the stimulation-evoked en masse LC activation than when considering only the activation of an individual ensemble), the associated cortical states should include more frequently the activated state and become more homogenous. We took advantage of our observation that ensemble pairs can sometimes become coactive (Fig. 4*B*). We assessed the cortical LFP spectrograms, as in Fig. 6*A* and *B*, but aligned spectrograms to spontaneously occurring coactivation times of ensemble pairs. A total of 199 ensemble pairs, which had a significant zero lag cross-correlogram peak, were simultaneously recorded with cortical LFP. In contrast to the four spectrogram types observed around the time of spontaneous activation of individual LC ensembles, clustering now revealed only two spectrogram types at the time points when ensemble pairs became synchronously coactive, suggesting a reduced diversity with respect to the individual ensemble analysis (Fig. 8). We refer to these two new clusters, which now characterize cortical power spectrograms around the synchronous activation of LC ensemble pairs, as clusters E and F. Cluster F is the stereotypical activated cortical state, which included more than half (101 of 199) of the ensemble pairs, suggesting a larger proportion of activated states associated with spontaneously occurring coactivation of different ensembles in comparison to when individual ensembles were active. Cluster E, on the other hand, is characterized by a relative decrease of spectral power across all bands (98 of 199 ensemble pairs). In sum, when two LC ensembles are coactive, such that LC population activity becomes more similar to en masse LC activation, the modulation of cortical state is more homogenous and presents a higher proportion of relative cortical power changes that are similar to the LC stimulation–evoked cortical state change (10–13).

## Discussion

Cortical states vary over a wide range and are in a tight relationship with many functions that are relevant to psychiatric disorders, such as sleep, arousal, perceptual ability, and reaction times. It is thus no surprise that there have been long-standing efforts to understand the neural factors contributing to cortical state fluctuations (1–3). However, the neuronal interactions that control cortical states remain largely elusive. Blocking out the effect of the external world (e.g., slow-wave sleep or anesthesia) has proven to be a successful approach for dissecting the spontaneous brain-internal neuronal interactions that control cortical state (34). These approaches have been used to demonstrate that the LC evokes transitions to, and maintenance of, a single and unitary activated state in the cortex (10–13). However, rather than studying the spontaneous emergence of cortical states due to LC activity, these studies have used electrical or optogenetic stimulation of the LC, which evokes en masse LC population activity.

Here, we considered the effect of spontaneously occurring events of LC ensemble activations. Using synthetic spike trains with different ground truth patterns of ensemble activity, we illustrate that NMF can detect the precise composition of ensembles, even when multiple ensembles occasionally coactivate or when neurons participate in multiple ensembles. By applying this methodology to LC population activity, we decomposed this spontaneous activity into multiple, separate neuronal ensembles. This enabled us to study quantitatively the spontaneous interactions between LC neurons and cortical states and contribute to long-standing efforts to understand how cortical state might be generated (1–3). We demonstrated that ongoing cortical state differs depending upon which LC ensemble was spontaneously active. We observed significant power variations around the activation times of LC ensembles. This result is consistent with the broad afferent input that the LC receives from the forebrain, which predicts that cortical states may affect individual LC ensembles and be affected by them. Importantly, these findings establish that LC ensembles do not simply evoke a stereotypical activated state in the cortex. Thus, the temporally diverse and largely nonoverlapping nature of spontaneous LC ensemble activations correspond to a diversity of cortical states. When LC ensemble pairs become synchronously coactive, which is a situation more similar to the highly synchronized and en masse LC population activation driven by LC stimulation (10–13), the diverse set of cortical states had a higher proportion of the stereotypical activated state that has been uniformly observed across these prior studies using LC stimulation. Coactivation of ensemble pairs, or multiensemble “collectives”, may provide transient global norepinephrine release. These global events may occur in response to specific, structured afferent input in specific moments during anesthesia, sleep, or wakefulness that demand more homogenous noradrenergic neuromodulation.

The types of cortical state modulations on a subsecond scale around spontaneous activations of LC ensembles that we documented here happened within the broader context of two longer (minutes) cortical states that occur during urethane anesthesia (35, 36). The predominant urethane-associated cortical state consists of slow waves and an LFP power spectrum that strongly resembles that of non–rapid eye movement slow-wave sleep. This slow-wave state is punctuated by continuously “activated” epochs that are devoid of slow waves, enriched with beta and gamma oscillations, and thus loosely resemble cortical activity during wakefulness. Our findings show that, even in the context of these continuous states, there are multiple substates that emerge when one examines cortical activity around brain-internal neuronal events, such as spontaneous LC ensemble activations. Using our analytic methodology during sleep or wakeful behavior may reveal many diverse cortical substates associated with LC ensemble activations. These studies will require novel tools for recording LC ensembles in nonanesthetized animals.

### Potential Neurophysiological Causes of the Diversity in Cortical State.

Neuromodulation of different forebrain regions may alter the brain-internal neuronal interactions that produce various cortical states. LC neurons are broadly projecting but also have localized projections to the forebrain and release a range of neurotransmitters (37, 38); therefore, LC ensembles that project to different forebrain neuronal networks could affect how those networks self-organize cortical states. When considering how distinct LC ensembles could promote different cortical states, two potential factors for future study are the diversity of ensemble neurochemical makeup and/or its projection profile. Given that the region in which we assessed cortical state (area 24a) receives projections from ∼61 to 65% of LC neurons in the rat (39, 40), it seems likely that most ensembles project to area 24a, and they should, therefore, produce a similar state change. Our finding to the contrary could be explained by the possibility that the neurochemical makeup of the LC neurons differs across ensembles and results in cortical state diversity. Another possibility is that despite most ensembles presumably sharing area 24a as a projection target, it is the other targets that are potentially not shared across ensembles that leads to LC ensemble–specific cortical states in 24a. According to this forebrain “network” perspective, LC ensembles associated with different cortical states could have divergent axon collaterals, which enable the ensembles to modulate distinct forebrain neuronal networks that are associated with different cortical states. Ensembles associated with the activated cortical state were smaller and fired more strongly. Smaller ensembles may have less diffuse projections and, with a higher spike rate, they may release larger amounts of norepinephrine or have increased likelihood of releasing other neurotransmitters that could be associated specifically with the activated cortical state.

### An LC Ensemble Code Enables Greater Diversity in Neuromodulatory Functions.

Behavioral and mental states fluctuate widely from moment to moment, and it has been known since the advent of EEG recordings that such diverse cognitive–behavioral states are associated with a multitude of cortical states (1–3). The brain-internal interactions that generate this large state space are still largely unknown. LC neurons were classically thought to modulate cortical and thalamic neuronal excitability level using noradrenergic “tone” (5–7), whereas the neuronal interactions that produce the cortical state are contained within the cortex and thalamus (41, 42). According to this standard view, the role of the LC has been to modulate or predispose cortico-thalamic circuits toward the activated cortical state (and predispose the organism toward wakefulness), but the actual neuronal interactions that select cortical state are between cortex and thalamus. However, this standard view of cortical state generation was developed using methods that artificially activated the LC neuronal population en masse using external stimulation. Here, we studied the spontaneously self-organized neuronal interactions that are internal to the brain to reveal that, in contrast to this classical thinking, distinct LC ensembles can promote diverse cortical states. Each ensemble in this small population of ∼1,600 brainstem noradrenergic neurons may individually be a key player in selecting ongoing cortical state from a multitude of possibilities. Thus, our findings shift the role of the LC from “modulator/promoter” of a single cortical state toward a “selector/controller” from a large subset of cortical states.

Our results imply that a single brainstem nucleus can perform different neuromodulatory functions by simply changing the groups of neurons that fire. Critically, these neuromodulatory dynamics can rapidly change on timescales relevant to flexible and ever-changing cognitive–behavioral states. The distinct LC ensembles with nuanced activation dynamics shown here substantially enrich the kind of functions that are currently attributed to brainstem nuclei.

## Materials and Methods

### Rats and Recording Procedures.

Male Sprague–Dawley rats (350 to 450 g) were used. Rats were anesthetized using an intraperitoneal injection of urethane (1.5 g/kg body weight). A detailed description of research subjects and recording procedures is in *SI Appendix*, *Materials and Methods* . All experiments were carried out with approval from the local authorities and in compliance with the German Law for the Protection of Animals in experimental research and the European Community Guidelines for the Care and Use of Laboratory Animals. A subset of the data was collected from rats used in a prior study (20). The LC electrode was targeted based on standard electrophysiological criteria and postexperiment administration of clonidine ( *SI Appendix*, *Materials and Methods* ). Single-unit type was defined by waveform duration (20).

### NMF Decomposition.

We used NMF (21) to decompose a matrix of the spike counts of all simultaneously recorded single units across time intervals. NMF linearly decomposes the matrix of the spike counts of the population of single units at each time interval as a sum across a set of nonnegative basis functions (modules) using nonnegative coefficients (21, 26, 27). A detailed description of its implementation is in *SI Appendix*, *Materials and Methods* . Briefly, NMF decomposes the population firing patterns across single units at each time interval (27): R=WH+residuals . $R\in Z_{+}^{T\;\times \;N}$ is the data matrix containing the spike counts of each of N single units binned into *T* time bins (with t being the index of each time bin). $H\in R_{+}^{K\;\times \;N}\;$ is the matrix containing the basis function, which has K spatial modules. Each module captures a different pattern of coactivity of the single units and can, therefore, be used to identify which neurons are active together and thus form ensembles. $W\in R_{+}^{T\;\times \;K}$ is the matrix containing the activation coefficients that describe the strength of recruitment of each module (and thus of each ensemble of coactive neurons) at each time interval. We binned spike counts at Δ*T* = 100 ms. The time resolution was selected based on our previous work reporting that pairs of LC single units are predominantly synchronized on a timescale of ∼100 ms or less (20). We also used ranges of Δ*T* from very small values (a few milliseconds) up to large values (a few seconds) and found that very small (≤20 ms) and very large (>1 s) bin sizes artificially identify either many modules each containing only one single active unit or one large ensemble containing all single units, respectively. We chose K for each rat by computing the amount of variance explained when varying K from its minimum (one) to its maximum possible value (the number of simultaneously recorded units).

### Auto- and Cross-Correlograms and Permutation Testing.

Auto-correlograms were calculated in a 1,000-ms time window using a 100-ms time bin. Cross-correlograms were calculated in a window of 2,000-ms with a bin size of 100 ms. To assess significance, the correlograms were compared to the upper and lower 1% bounds of 1,000 surrogate correlograms, which were created by jittering the activation times uniformly between ±1 s. These procedures are as in ref. 20.

### Definition of the Synchrony Index.

The degree of synchrony between ensemble pairs that had a significant peak at time 0 in the cross-correlogram was measured using a synchrony index:

$$\text{synch}=\left(\frac{2\times c_{ij}}{\tau_{i}+\tau_{j}}\right)\times 100,$$

where $c_{ij}$ is the number of times the two ensembles are coactive, and $\tau_{i},\tau_{j}$ are the number of active times for each ensemble.

### LC Ensemble Activation–Aligned Averaged LFP Spectrogram and BLP Modulations.

For each detected ensemble activation event, spectral analysis used data comprised of 400 ms before the beginning of ensemble activation, ran through a fixed 100 ms meant to approximate the ∼100-ms average duration of ensemble activation, and continued for 400 ms after the end of ensemble activation. This window was chosen because it is the largest one, according to our data (Figs. 2*C* and 4*B*), for which it is unlikely that multiple ensembles were coactive during this window. Spectrograms were computed using the multitaper method with three tapers and time bandwidth product of 5 in a 200-ms window shifted in 10-ms steps. The spectral resolution obtained this way was ∼4 Hz. The 200-ms sliding window size allows extracting from the analysis window an estimation of spectrograms in windows whose center falls between 300 ms before to 400 ms after ensemble activation onset, which were thus used as ranges for spectrogram plots in Fig. 6 and *SI Appendix*, Fig. S3.

Importantly, it should be noted that this short time window necessitated a low-frequency resolution (∼4 Hz), and this coarse frequency resolution may be insufficient to resolve, in a graded and smooth way, some transitions between frequency bands such that power changes sharply rather than gradually across frequencies. Although wavelet transformation would provide higher frequency and time resolution in comparison to the multitaper method, it would contain too much data for reliable clustering given the number of ensemble activation times. Therefore, the multitaper method was chosen because of the need for robust clustering down the analysis pipeline.

BLP was computed by filtering the LFP backward and forward to avoid phase shifts (filtfilt function, MATLAB) using a third-order Butterworth filter, taking the absolute value of the Hilbert transform of the filtered signal, and finally smoothing them with a 200-ms Gaussian window (the value of the smoothing was chosen to match the size of the sliding time window used for spectrograms). For consistency, we plotted BLP and spectrograms using the same perievent activation window ranges.

For each ensemble, we first averaged BLPs and spectrograms across all events. To compare spectrograms and BLPs across ensembles, we normalized them to a spectral modulation index defined by the ratio between the difference and sum of the spectral value at each time point and frequency bin and its time average in the frequency bin over the entire perievent window. This quantity at each time point and frequency bin can take values between −1 and 1 and quantifies the relative changes of power around the time-averaged power in each frequency bin in the main text. Spectra were clustered using a *k*-means algorithm. Significance of spectrogram modulations of cluster centers (Fig. 6*A*) at each specific time frequency point was computed as significance of deviation from a zero median value using a two-tailed Wilcoxon signed rank and correcting the so obtained *P* values for multiple comparison at a false discovery rate of *q* = 0.05. Full details of all procedures are reported in *SI Appendix*, *Materials and Methods* .

## Supplementary Material

Supplementary File

## Data Availability

All data (single-unit spike times and local field potentials) and the analysis code have been deposited in a publicly accessible database, Gnode (https://doi.gin.g-node.org/10.12751/g-node.3rrsh5/) (43). Previously published data were used for this work (20).
