# Supplementary material for: Distinct ensembles in the noradrenergic locus coeruleus are associated with diverse cortical states
Source: Proc Natl Acad Sci U S A. 2022 Apr 29;119(18):e2116507119. doi: 10.1073/pnas.2116507119 (PMC9170047; doi:10.1073/pnas.2116507119)
Supplement: Supplementary File [file pnas.2116507119.sapp.pdf]

PNAS
www.pnas.org

Supplementary Information for
Distinct ensembles in the noradrenergic locus coeruleus are associated with diverse cortical states

Shahryar Noei, Ioannis S. Zouridis, Nikos K. Logothetis, Stefano Panzeri, Nelson K. Totah

Nikos K. Logothetis
Email: nikos.logothetis@icpbr.ac.cn

Nelson K. Totah
Email: nelson.totah@helsinki.fi

Stefano Panzeri
Email: s.panzeri@uke.de

This PDF file includes:

Supplementary text
Figures S1 to S5
References for supplementary text

Supplementary Information Text

Materials and Methods

Recording procedure and signal acquisition.

All experiments were carried out with approval from the local authorities and in compliance with the German Law for the Protection of Animals in experimental research and the European Community Guidelines for the Care and Use of Laboratory Animals. Male Sprague-Dawley rats (350 - 450 g) were used (specific pathogen free, Charles River Laboratories, Sulzfeld, Germany). They were pair housed. Experiments were carried out during the active period of the rats, which were housed on a light cycle of 08:00 to 20:00 darkness. A sub-set of the data were collected from rats used in a prior study (1).

Neuronal recordings were made under urethane anesthesia, a widely used model for studying cortical state transitions evoked by LC stimulation (2, 3). To date, recordings of many LC single units simultaneously in any awake organism with multi-electrode probes has been an intractable problem due to brainstem movement associated with body movement, thus necessitating the use of anesthesia to investigate the relationship between LC ensemble activity and cortical state.

Rats were anesthetized using an intra-peritoneal (i.p.) injection of urethane at a dose of 1.5 g/kg body weight (Sigma-Aldrich, U2500). Surgical procedures were as described in prior work (1). Electrodes targeted the LC and the prelimbic division of the medial prefrontal cortex. The coordinates for LC were 4.0 mm posterior from lambda, 1.2 mm lateral from lambda, and approximately 6.0 mm ventral from the brain surface (implanted at a 15 deg posterior angle). The coordinates for the cortex were 3.0 mm anterior and 0.8 mm lateral

from bregma and 3.0 mm ventral from the brain surface. The LC electrode was targeted based on standard electrophysiological criteria (1). Briefly, we inserted a 32-electrode array and monitored the neural activity on all 32 channels during insertion. We advanced the array ventrally until the biphasic response (i.e., excitation, followed by auto-inhibition and lateral inhibition) to noxious foot shock (5.0 mA biphasic square pulse, 0.5 msec duration) occurred clearly on all 32 channels. If the biphasic response was not observed on all channels, then the penetration was not the LC and was not included in the study. According to this criterion, we found that it was possible to have all electrodes (spanning 275 um in dorso-ventral axis) within the LC core, which spans 500 um dorso-ventrally. Our array was therefore advanced to the interior of the LC and could not span the entire extent of the LC. In addition to biphasic response to noxious stimuli, LC single units were characterized by a wide spike waveform, low firing rate, and long inter-spike intervals. At the end of the recording, we administered clonidine (0.05 mg/kg) i.p. (Sigma-Aldrich, product identification: C7897) to confirm cessation of noradrenergic neuron spiking across the entire recording array. We also verified LC targeting in most experiments using histological examination of coronal sections (50 um thick) that were stained for Cresyl violet or a DAB and horse radish peroxidase reaction with hydrogen peroxide to visualize an antibody against tyrosine hydroxylase, as shown in prior work (1).

The LC was recorded using a multi-channel silicon probe (NeuroNexus, Model: A1x32-Poly3-10mm-25s-177-A32). The impedance of the electrodes was ~1.0 to 2.0 MOhm. Cortical local field potentials were recorded using a single tungsten electrode with an impedance of 200 – 800 kOhm (FHC). A chlorided silver wire inserted into the neck muscle was used as a ground. Electrodes were connected to a pre-amplifier (in-house constructed) via low noise cables. Analog signals were amplified (by 2000 for LC and 500 for cortex) and filtered (8 kHz low pass, DC high pass) using an Alpha-Omega multi-channel processor (Alpha-Omega, Model: MPC Plus). Signals were then digitized at 24 kHz using a data acquisition device (CED, Model: Power1401mkII).

NMF decomposition of population spike trains into coactive ensembles.

We used non-negative matrix factorization (NMF) (4) to decompose a matrix of the spike counts of all simultaneously recorded single units across time intervals. NMF linearly decomposes the matrix of the spike counts of the population of single units at each time interval as a sum across a set of non-negative basis functions (modules) using non-negative coefficients (4–6). The non-negativity constraint is useful for obtaining sparse representations and it is particular suitable for decomposing population spike count at different time intervals, which are always non-negative. Previous work has shown that the NMF of population spike trains provides a robust decomposition whose basis functions can be biologically interpreted as a set of the firing patterns of the single units that are coactive (i.e., an ensemble) and the coefficients quantify the relative strength of recruitment of each ensemble firing pattern at any given time (5).

We employed an NMF decomposition that we have previously termed “space only NMF” because it decomposes the population firing patterns across single units at each time interval (5):

$$\mathrm{R} = \mathrm{WH} + \mathrm{residuals}$$

$\mathrm{R} \in \mathbb{Z}_{+}^{T \times N}$ is the data matrix containing the spike counts of each of N single units binned into T time bins (with t being the index of each time bin). $\mathrm{H} \in \mathbb{R}_{+}^{K \times N}$ is the matrix containing the basis function, which has K spatial modules. Each module captures a different pattern of coactivity of the single units and can, therefore, be used to identify which neurons are active together and thus form ensembles. $\mathrm{W} \in \mathbb{R}_{+}^{T \times K}$is the matrix containing the activation coefficients that describe the strength of recruitment of each module (and thus of each ensemble of coactive neurons) at each time interval. The residuals express the error in the reconstruction of the original population spike train matrix. We computed the decomposition using the multiplicative update rule to minimize the Frobenius norm between the original and the reconstructed data (4). Note that the use of the Frobenius norm assumes that the residuals have a Gaussian white noise structure.

One free parameter of the analysis is the temporal resolution of the time binning, ΔT. We binned spike counts at ΔT = 100 ms. The time resolution was selected based on our

previous work reporting that pairs of LC single units are predominantly synchronized on a timescale of approximately 100 ms or less (1). We also used ranges of ΔT from very small values (a few ms) up to large values (a few seconds) and found that very small (<= 20 ms) and very large (> 1 s) bin sizes artificially identify either many modules each containing only one single active unit or one large ensemble containing all single units, respectively.

The second free parameter of the NMF analysis is the number of different modules, K, which were chosen for computing the decomposition. Following established procedures (5, 6), we chose K for each rat by computing the amount of the variance explained by the decomposition when varying K from its minimum possible value (one) to its maximum possible value (the number of simultaneously recorded single units). An elbow in this plot indicates a point of diminishing returns for including more modules. We thus chose the number of modules as the smallest K in the elbow region of this curve for which the decomposition reconstructed at least 60% of the variance of the original spike train data. Given that the NMF decomposition may have local maxima in the variance explained (or equivalently local minima in error reconstruction), after selecting K, we repeated the decomposition five times using this K and used randomly chosen initialization conditions on each repetition. The selected K was used if all solutions had a high degree of stability across these five random initializations. Stability was assessed by checking the repeatability of clustering in comparison to randomly assigning single units to ensembles. The degree of stability was computed as follows. We hard clustered the data to assign each single unit to one and only one ensemble by dividing each column of H by its maximum and removing the values below 1. From these data we then measured the stability across the five decompositions using the Rand Index (7). We compared the average of the Rand index for each animal with 100 repetitions of the five random clustering. The average Rand Index was always greater than the top 5% of the distribution of mean Rand Indices resulting from random clustering. Therefore, NMF decomposition produced meaningful and repeatable ensembles. Among those random initializations, the final decomposition reported in the analyses was chosen as the one leading to the maximum variance explained.

The modules detected by NMF provide a pattern of coactivation of different single units and the activation coefficients measure the strength of recruitment of each module at any given time. From these data, we used a threshold-crossing of the coefficients to define when ensembles were active and which single units were active in the ensemble. In order to perform the thresholding, we first normalized the columns of H to the minimum and maximum and then set a threshold based on the distribution of coefficients. Single units within a module were defined as an ensemble of coactive single units if their corresponding element of H crossed the initial big peak of the histogram of the distribution of coefficient values for that rat (which usually corresponded to 95th percentile). Coefficients below this value were set to zero and values above the threshold were set to one. In the resulting binary version of the matrix, H, a value of 1 represented spatial modules corresponding to a single unit belonging to an ensemble.

The columns of the W matrix correspond to a set of activation coefficients representing the strength of recruitment of each module at any given time interval. We thresholded these continuous values into binary values using the same method explained above for the spatial modules. The binary version of the matrix, W, was used to determine whether an ensemble is active or not in each time bin.

The evaluation of physical clustering of ensembles according to location on the recording array.

To assess whether single units within an ensemble tended to cluster on the recording array, we measured the pairwise distance between the units within each ensemble. The location of each unit was assigned to the electrodes on which the maximal waveform was recorded. Euclidian pairwise distances of the units inside each ensemble were calculated.

Spike train simulation

We compared the NMF algorithm with previous work that used graph-theoretic community detection of subsets of co-active neurons from the time-averaged correlations between LC cell-pairs (1), we generated three different sets of spike trains. Each set of spike trains had unique ensemble dynamics as a ground-truth. Spike trains were generated using Poisson

Process, with time varying rate every 100 ms. The baseline rate was randomly sampled from a Gaussian distribution with a mean of 1 and standard deviation of 0.1. For the periods of co-activation, the rate was increased by a signal to noise ratio sampled from a Gaussian with a mean of 1.5 and standard deviation of 0.1. After the generation of the spike trains, we binned and counted the spikes every 100 ms. This matrix was fed to NMF for extraction of spatial and temporal modules. Pairwise spike count correlation was calculated using Pearson correlation on the spike count matrix. A graph was made on the correlation matrix with each unit as a node and the links with the nodes was drawn only for significant correlations. The significance of the correlation was assessed by comparing to 1,000 surrogate correlations generated by shuffling spike counts randomly in time. The graph was then used as an input for the Louvain community detection as described in prior work (1).

The assignment of single unit types in the ensembles.

Single unit type was defined by waveform duration, as in prior work (1). We determined if single units of the same type were more likely than chance to belong to an ensemble by computing the exact probability of having ensembles of the same single unit type under the null hypothesis of random assignment. These probabilities were computed by the means of repetition of random sampling (assembling) without replacement. The number of units in the sample was fixed to the number of single units in the ensemble. The number of repetitions for each rat was the number of ensembles that were empirically found by NMF to consist of only one type of single unit.

Calculation of cross-correlograms between pairs of ensembles.

Interactions between pairs of ensembles were measured using cross-correlograms between their time-dependent activation coefficients. Cross-correlograms were calculated in a window of 2000 ms with a bin size of 100 ms. The cross-correlograms were compared to 1,000 surrogate cross-correlograms by jittering the activation times uniformly between ±1000 ms. Significant excitatory interactions were those that had cross-correlogram bins

which crossed the upper 1% of pairwise coincidental activations observed in the surrogate data.

A synchrony index was used to measure the degree of synchrony between the ensemble pairs that show significant zero-lag cross-correlogram peak. We calculated the synchrony index by dividing the number of co-activations of the two ensembles by the sum of total number of activations of each of the ensembles.

LC ensemble activation-aligned averaged LFP spectrogram and BLP modulations. For each detected ensemble activation event, we used for spectral analysis data comprising 400 ms before the beginning of ensemble activation, 100 ms of ensemble activation, and 400 ms after the end of ensemble activation. This window was chosen because it is the largest one, according to our data (**Figure 2C, Figure 4B**), for which it is unlikely that multiple ensembles were co-active during this window. Spectrograms were computed using the multitaper method with 3 tapers and time bandwidth product of 5 in a 200 ms window shifted in 10 ms steps. The spectral resolution obtained this way was ~4 Hz. The 200 ms sliding window size allows extracting from the analysis window an estimation of spectrograms in windows whose center falls between 300 ms before to 400 ms after ensemble activation onset, which were thus used as ranges for spectrogram plotting in **Figure 6** and **Figure S3**. Band-limited power (BLP) was computed by filtering the LFP backward and forward to avoid phase shifts (filtfilt function, MATLAB) using a 3rd order Butterworth filter, then taking the absolute value of the Hilbert transform of the filtered signal, and finally smoothing them with a 200 ms Gaussian window (the value of the smoothing was chosen to match the size of the sliding time window used for spectrograms). For consistency, we plotted BLP and spectrograms using the same peri-event activation window ranges.

For each ensemble, we first averaged BLPs and spectrograms across all events. To compare spectrograms and BLPs across ensembles, we normalized them to a spectral modulation index defined by the ratio between the difference and sum of the spectral value at each time point and frequency bin and its time-average in the frequency bin over the entire peri-event window. This quantity at each time point and frequency bins can take values between -1

and 1 and quantifies the relative changes of power around the time-averaged power in each frequency bin in the main text. Spectra were clustered using a k-means algorithm.

To illustrate the significance of BLP modulations of individual ensembles, in Figure 5B we compared the scatter plot of BLP modulations of real data against those that were obtained by computing BLPs aligned to randomly shuffled ensembles activation times (keeping the number of activations the same as in the real data but shuffling their times randomly). We found (Figure 5B) that shuffled times modulations were smaller than those obtained with real ensemble activation times.

Spectrogram clustering.

The set of ensemble activation-triggered spectral modulations were clustered to recapitulate the main trends observed within the diversity of LC ensemble activation-triggered cortical spectrograms. The clustering was performed using the k-means algorithm (9). The k-means algorithm requires specifying a choice for the number of possible clusters and for the mathematical function used to compute the distance between the different spectrograms. We tried various definitions of distance functions (Pearson Correlation, Euclidean distance, cosine, and cityblock), and we chose Pearson correlation as distance function because it gave higher averaged silhouette values (10), which suggests cleaner clustering. Clustering was done on the spectrogram modulation values and not on those thresholded for significance. We clustered the spectral modulation into k=4 clusters. This number of clusters was selected because it corresponded to the elbow point (defined as the first point in which the error drops below 5%) of the curve quantifying the normalized clustering error (error divided by the maximum error) as a function of the selected number of clusters. The error in the k-means clustering was computed as sum of the distances of each data point to their respective cluster centroid. We assessed the significance of the clustered spectral modulations at each time and frequency shown in Figure 6A by pooling the spectral modulations of all ensembles in each cluster and comparing the median of the population at each point against zero using a two-tailed Wilcoxon signed rank test. The p-values were corrected for multiple comparisons using Benjamini's & Hochberg's method for false discovery rate at q = 0.05 (11).

The above analysis was done taking for clustering all spectral modulations obtained in correspondence of a detected activation of one or more ensembles. We performed a further control analyses in which we clustered only the subset of the spectral modulations during coactivation of ensemble-pairs. The clustering procedure for this control analysis was identical to the one reported above but selected a number of clusters (corresponding to the elbow point of the error curve) equal to 2 clusters.

Supplemental Information

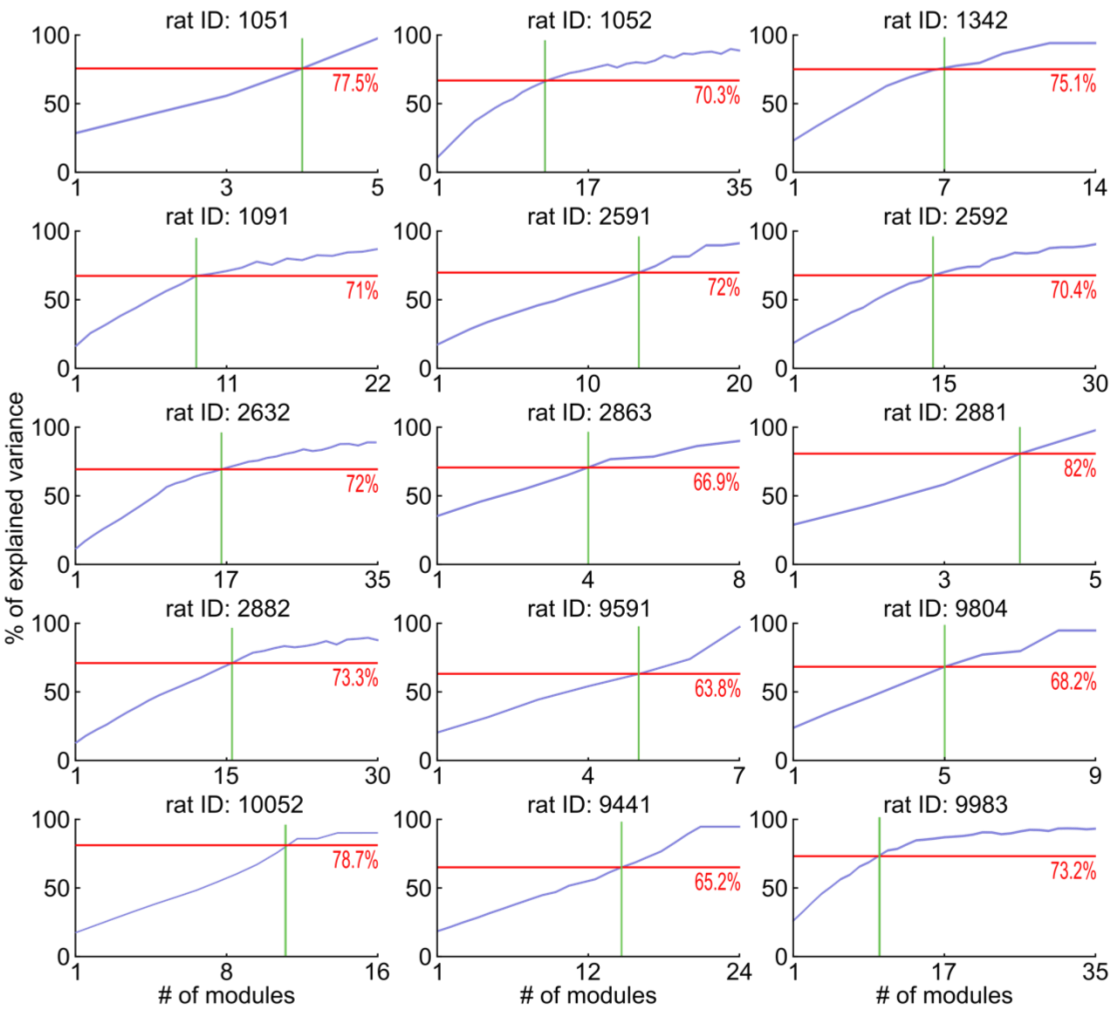

Fig. S1. Data underlying the choice of the optimal number of modules (K) in each rat. Each panel depicts the percentage of explained variance versus the number of the modules for each rat. Solid green lines show the number of selected modules based on the criteria of first elbow after at least 60% of variance is explained. The dotted red lines show the amount of the explained variance at the selected number of modules.

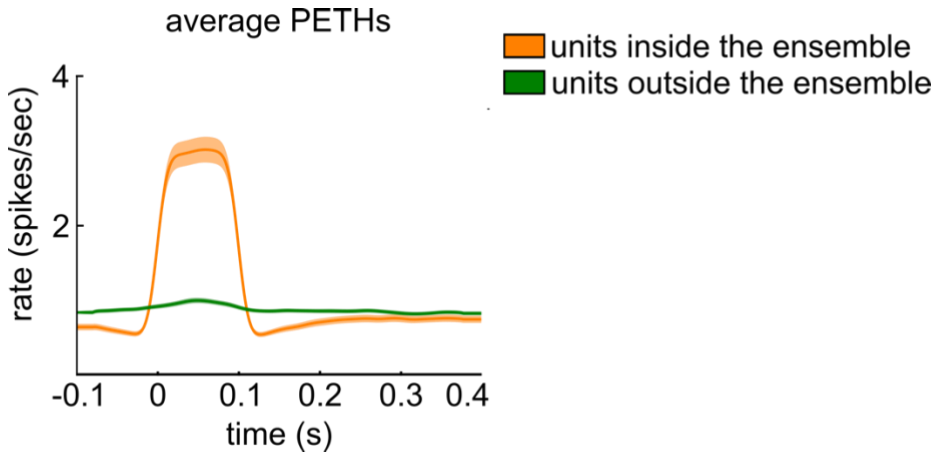

Fig. S2. Spike rates of units inside and outside LC ensembles around the time of spontaneous LC ensemble activation. Average PETHs over all the ensembles (N = 146). The zero time on the x-axis is the ensemble active time. Line and shaded area report the mean and SEM across ensembles, respectively.

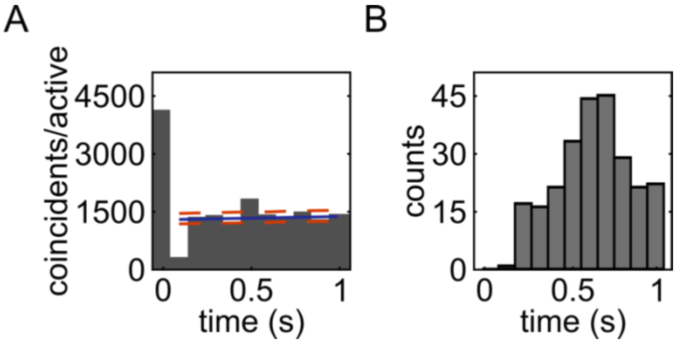

Fig. S3. Ensemble auto-correlogram peaks. (A) The histogram plots the auto-correlogram (time binning of 100 ms) of the activation time course of an example ensemble (#3 from rat 1091). Significant peaks were defined as those that had auto-correlogram values above the 99th percentile (upper dashed orange line) of the surrogate distributions of correlogram values computed by randomly jittering ensemble activation times. The solid blue line shows the average of the surrogate correlograms. **(B)** Histogram of the number of significant auto-correlogram peaks as a function of time lag. 117 out of 146 ensembles had a significant peak.

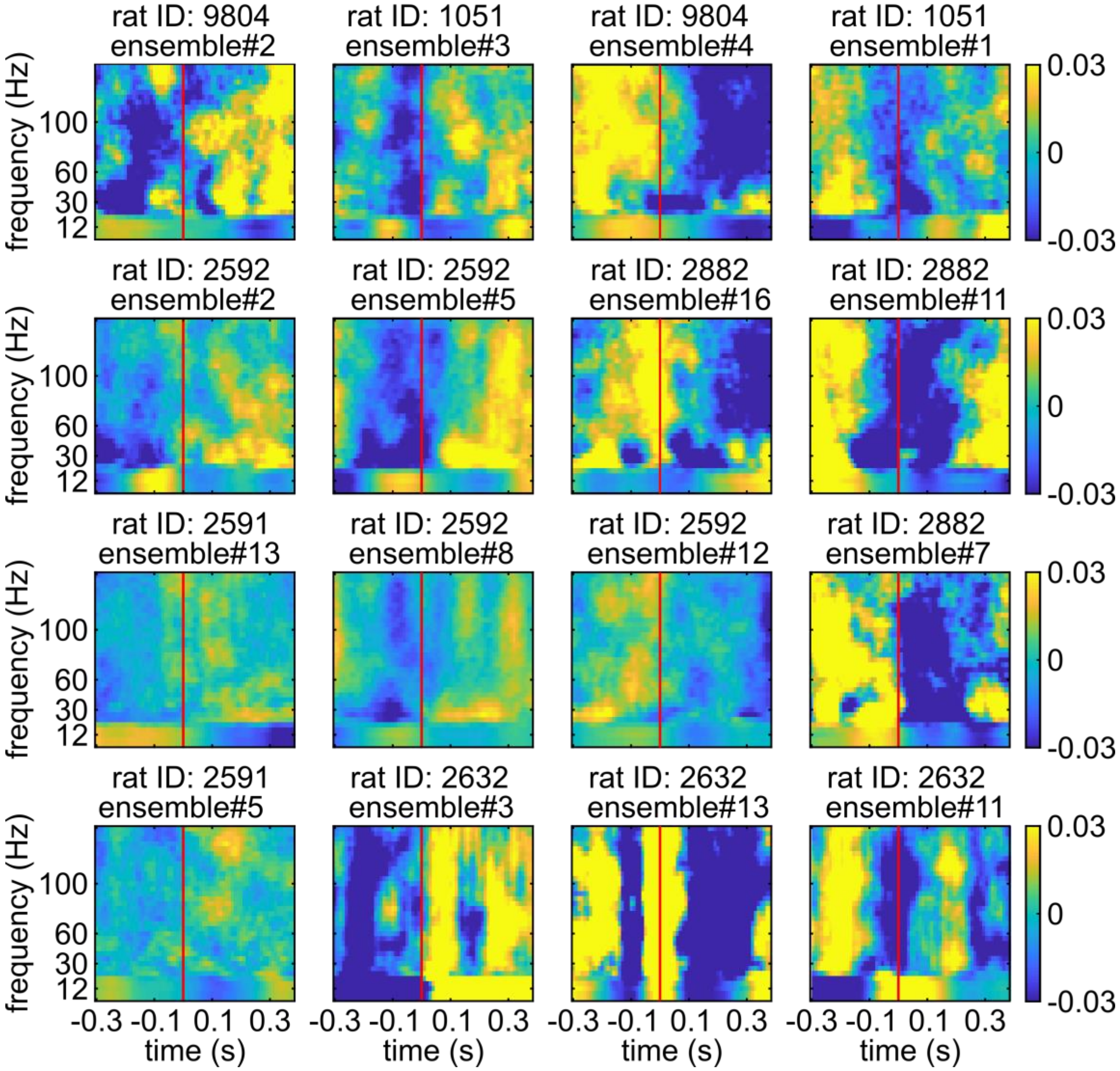

Fig. S4. Examples of cortical LFP power spectrograms aligned to spontaneous activations of individual LC ensembles. Examples from 12 different ensembles illustrate the diverse cortical states which occur around the time of ensemble activation. The examples are shown in 4 columns, with each column reporting spectrograms that were assigned respectively to the clusters A, B, C, and D shown in the Figure 6 in the Main Text.

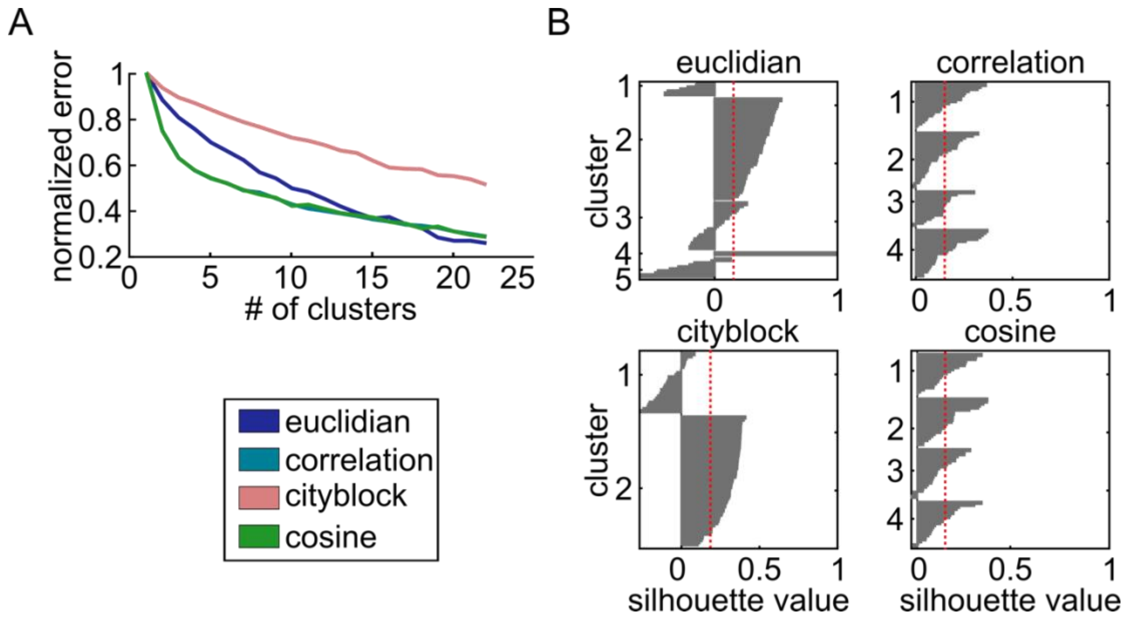

Fig. S5. The result of analyses supporting the determination of the best criteria for spectral clustering. (A) The normalized error (error divided by the maximum error) of the k-means clustering of the ensemble activation-aligned spectra versus the number of clusters. Four different distance measures were assessed and each is plotted in a different color. **(B)** Each panel shows the result of the silhouette analyses on the chosen number of clusters for four different distance measures. The optimal distance was selected based on both the uniformity in each cluster (the width of the bar plots) and the average silhouette value (the dashed red line).

SI References

1. N. K. Totah, R. M. Neves, S. Panzeri, N. K. Logothetis, O. Eschenko, The Locus Coeruleus Is a Complex and Differentiated Neuromodulatory System. *Neuron* 99, 1055-1068.e6 (2018).

2. A. Marzo, N. K. Totah, R. M. Neves, N. K. Logothetis, O. Eschenko, Unilateral electrical stimulation of rat locus coeruleus elicits bilateral response of norepinephrine neurons and sustained activation of medial prefrontal cortex. *J Neurophysiol* 111, 2570–2588 (2014).

3. R. M. Neves, S. van Keulen, M. Yang, N. K. Logothetis, O. Eschenko, Locus Coeruleus phasic discharge is essential for stimulus-induced gamma oscillations in the prefrontal cortex. *J Neurophysiol* 119, jn.00552.2017 (2018).

4. D. D. Lee, H. S. Seung, Learning the parts of objects by non-negative matrix factorization. *Nature* 401, 788–791 (1999).

5. A. Onken, *et al.*, Using Matrix and Tensor Factorizations for the Single-Trial Analysis of Population Spike Trains. *Plos Comput Biol* 12, e1005189 (2016).

6. A. H. Williams, *et al.*, Unsupervised Discovery of Demixed, Low-Dimensional Neural Dynamics across Multiple Timescales through Tensor Component Analysis. *Neuron* 98, 1099-1115.e8 (2018).

7. W. M. Rand, Objective Criteria for the Evaluation of Clustering Methods. *J Am Stat Assoc* 66, 846 (1971).

8. P. Mitra, H. Bokil, *Observed Brain Dynamics* (Oxford University Press, 2007) https:/doi.org/10.1093/acprof:oso/9780195178081.001.0001.

9. D. Arthur, S. Vassilvitskii, k-means++: The Advantages of Careful Seeding. 1027–1035 (2007).

10. P. J. Rousseeuw, Silhouettes: A graphical aid to the interpretation and validation of cluster analysis. *J Comput Appl Math* 20, 53–65 (1987).

11. Y. Benjamini, Y. Hochberg, Controlling the False Discovery Rate: A Practical and Powerful Approach to Multiple Testing. *J Royal Statistical Soc Ser B Methodol* 57, 289–300 (1995).
